# Statistical Comparison of Classifiers Applied to the Interferential Tear Film Lipid Layer Automatic Classification

**DOI:** 10.1155/2012/207315

**Published:** 2012-04-05

**Authors:** B. Remeseiro, M. Penas, A. Mosquera, J. Novo, M. G. Penedo, E. Yebra-Pimentel

**Affiliations:** ^1^Departamento de Computación, Universidade da Coruña, Campus de Elviña S/N, 15071 A Coruña, Spain; ^2^Departamento de Electrónica y Computación, Universidade de Santiago de Compostela, Campus Universitario Sur, 15782 Santiago de Compostela, Spain; ^3^Escuela de Óptica y Optometría, Universidade de Santiago de Compostela, Campus Universitario Sur, 15782 Santiago de Compostela, Spain

## Abstract

The tear film lipid layer is heterogeneous among the population. Its classification depends on its thickness and can be done using the interference pattern categories proposed by Guillon. The interference phenomena can be characterised as a colour texture pattern, which can be automatically classified into one of these categories. From a photography of the eye, a region of interest is detected and its low-level features are extracted, generating a feature vector that describes it, to be finally classified in one of the target categories. This paper presents an exhaustive study about the problem at hand using different texture analysis methods in three colour spaces and different machine learning algorithms. All these methods and classifiers have been tested on a dataset composed of 105 images from healthy subjects and the results have been statistically analysed. As a result, the manual process done by experts can be automated with the benefits of being faster and unaffected by subjective factors, with maximum accuracy over 95%.

## 1. Introduction

The ocular surface is covered with the tear film, which was classically described by Wolff [1] as a three-layered structure, comprising an anterior lipid layer, an intermediate aqueous layer, and a deep mucin layer. The tear film provides a smooth optical surface by compensating for the microirregularities of the corneal epithelium and plays an essential role in the maintenance of ocular integrity by removing foreign bodies from the front surface of the eye.

The lipid layer is an essential component of the tear film and its principal function is to prevent the evaporation of tears during the interblink period and enhance the stability of the tear film. Thus, a deficiency of this layer, in the absence of an adequate increase of tear production by lachrymal glands gives rise to the *evaporative dry eye syndrome* [2], a disease which affects a wide sector of the population, especially among contact lens users, and worsens with age.

**Figure 1:**
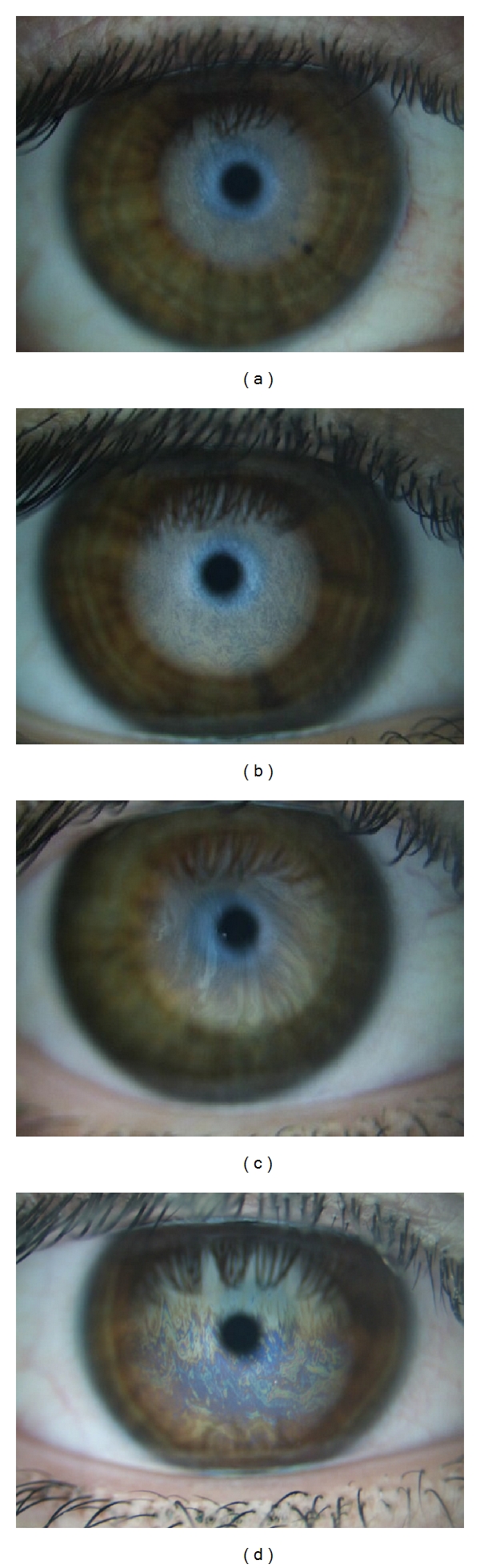
Lipid layer interference patterns: (a) Open meshwork. (b) Closed meshwork. (c) Wave. (d) Colour fringe.

The lipid layer thickness can be evaluated through the observation of the interference phenomena, since the colour and shape of the observed patterns reflect the layer thickness. Thicker lipid layers (≥90 nm) show colour and wave patterns while thinner lipid layers (≤60 nm) are more homogeneous. The Tearscope-plus [3], designed by Guillon, is an instrument which enables tear film assessment within the clinical and the laboratory setting and provides qualitative and semiquantitative information regarding the thickness and behaviour of the lipid layer in normal, pathological, and contact lens-wearing eyes. The lipid structure was categorised by Guillon based on the appearance of the specularly reflected lipid layer. Guillon defined five main grades of lipid layer interference patterns in increasing thickness [4]: open meshwork, closed meshwork, wave, amorphous, and colour fringe. The amorphous category has not been included in this study due to the lack of images from this category in the clinical image dataset used for validation. The four categories considered are illustrated in Figure 1. Open meshwork pattern (Figure 1(a)) represents a 13–15 nm lipid layer thickness with a grey appearance of low reflectivity, sparse, meshwork pattern faintly visible following the blink. It means a very thin, poor, and minimal lipid layer stretched over the ocular surface. Closed meshwork pattern (Figure 1(b)) refers to a lipid layer thickness of 30–50 nm. This is a more compact meshwork pattern with grey appearance of average reflectivity and more lipid than open meshwork. This pattern represents a normal lipid layer that could be suitable for contact lens wear. The wave pattern (Figure 1(c)) is the most common lipid layer which is related to a 50–80 nm lipid layer thickness. Its appearance is of vertical or horizontal grey waves of good visibility between blinks. It means an average tear film stability suitable for contact lens wear. Finally, the colour fringe pattern (Figure 1(d)) represents a thicker lipid layer with a mix of colour fringes well spread out over the surface. Its appearance is of discrete brown and blue well-spread lipid layer interference fringes superimposed on a whitish background. The thickness ranks 90–140 nm and represents a regular, very full lipid layer. This pattern indicates a good candidate for contact lens wear but with possible tendency for greasing problems or lipid deposits if a contact lens is fitted.

The classification of the lipid layer thickness is a difficult clinical technique, especially with thinner lipid layers that lack colour fringes and other morphological features, and is affected by the subjective interpretation of the observer. Some techniques have been designed to objectively calculate the lipid layer thickness, where a sophisticated optic system was necessary [5] or an interference camera evaluated the lipid layer thickness by analysing only the interference colour [6]. In a previous paper [7], we have demonstrated that the interference phenomena can be characterised as a colour texture pattern with classification rates over 95%. The classification is automatic, saving time for experts, who do this time-consuming task by hand, and eliminating the subjectivity of the manual process.

In that previous work, we generated a wide set of feature vectors using different texture analysis methods in three colour spaces and we classified them using support vector machines (SVMs). In this study, we perform several experiments using a wide set of machine learning algorithms and analyse their behaviour, in order to statistically determine which classifier works better in the problem at hand and use it in our future work.

This paper is organised as follows: Section 2 explains the methodology for the automatic classification of the lipid layer; Section 3 compares the experimental results; finally, Section 4 exposes our conclusions and future work.

## 2. Methodology

Our methodology for the tear film classification consists of four stages. The first stage entails the acquisition of the input image and has been carried out with the Tearscope-plus [3] attached to a Topcon SL-D4 slit lamp [8] and a Topcon DV-3 digital video camera [9]. The slit lamp's magnification was set at 200X and the images were stored via the Topcon IMAGEnet i-base [10] at a spatial resolution of 1024 × 768 pixels per frame in RGB. Since the tear lipid film is not static between blinks, a video has been recorded and analysed by an optometrist in order to select the best images for processing. Those images were selected when the tear lipid film was completely expanded after the eye blink.

**Figure 2:**
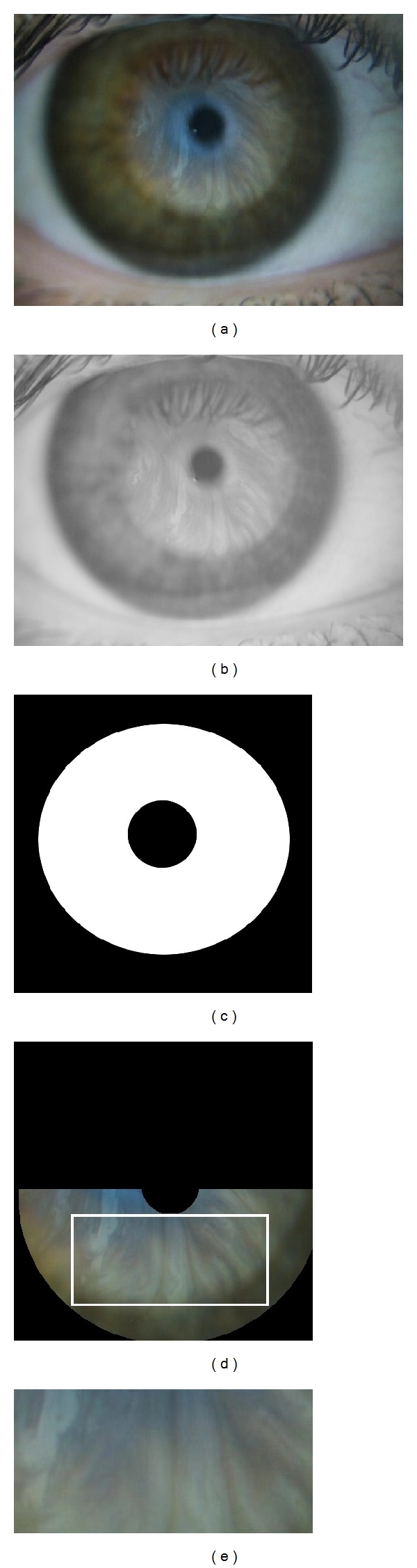
(a) Input image in RGB. (b) Luminance component L of the input image transformed to the Lab colour space. (c) Template used to locate the region of interest. (d) Subtemplate and region of interest. (e) ROI of the input image.

The input images, as depicted in Figure 1, include several areas of the eye that do not contain relevant information for the classification; such as the sclera, eyelids, and eyelashes. Experts that analyse these images usually focus on the bottom part of the iris, because this is the area where the tear can be perceived with better contrast. This forces a preprocessing step [11], which corresponds to the second stage of the methodology, aimed at extracting the region where the lipid tear film classification takes place, called *region of interest* (ROI). Our acquisition procedure guarantees that this region corresponds to the most illuminated area of the image. Thus, in order to restrict our analysis to the illumination, we transform the input image in RGB to the Lab colour space [12] and only use its luminance component L. Then, we select the region of the image with maximum normalised cross-correlation between the L component and a template from a previously generated set, composed of several templates that cover the various shapes the ROI can have (see Figure 2 for an example of this stage).

 After extracting the regions of interest, the next step entails analysing their low-level features. Colour and texture seem to be two discriminant features of the Guillon categories. Thick lipid layers show clear patterns while thinner layers are more homogeneous. Also, since some categories show distinctive colour features, we have analysed the low-level texture features not only in grayscale but also in Lab [12] and in RGB, making use of the opponent colour theory [13]. Finally, the last stage classifies the images into the categories previously mentioned. In the following sections, we explain these two main stages in detail.

### 2.1. Texture Analysis

Our textural features are extracted by applying five popular texture analysis methods [14]: *Butterworth filters*, *the discrete wavelet transform*, *cooccurrence features*, *Markov random fields*, and *Gabor filters*. First, we explain all these methods in depth using grayscale images, then we introduce the two colour spaces considered.

#### 2.1.1. Butterworth Filters

A Butterworth bandpass filter [15] is defined as

(1) $$f\left(\omega \right)=\frac{1}{1+{\left(\left(\omega -\omega_{c}\right)/\omega_{0}\right)}^{2n}},$$

where *n* is the order of the filter, *ω* the angular frequency, *ω*_0_ the cutoff frequency, and *ω*_*c*_ the centre frequency. The order *n* of the filter defines the slope of the decay; the higher the order, the faster the decay.

In the present work, we have used a bank of Butterworth bandpass filters composed of 9 second-order filters, with bandpass frequencies covering the whole frequency spectrum [16]. The filter bank maps each input image into 9 result images, one per frequency band.

In order to classify the input images, we must assign each of them a feature vector. To generate this vector, we have first normalised each frequency band results separately and the computed histograms of its output images. Those histograms concentrated most of the information in the lower bins, which made their comparison difficult. In order to increase the relevance of the differences among lower values, we computed uniform histograms with nonequidistant bins [16].

Since we are using 16 bin histograms, our feature vectors contain 16 components. 

#### 2.1.2. The Discrete Wavelet Transform

Mallat [17] was the first to show that wavelets formed a powerful basis for multiresolution theory, defining a mathematical framework which provides a formal, solid, and unified approach to multiresolution representations. This wavelet paradigm has found many applications in signal and image processing, such as texture analysis.

The discrete wavelet transform generates a set of wavelets by scaling and translating a *mother wavelet*, which is a function defined both in the spatial and frequency domain, that can be represented in 2D as [18]

(2) $$\phi^{a,b}\left(x,y\right)=\frac{1}{\sqrt{a_{x}a_{y}}}\phi \left(\frac{x-b_{x}}{a_{x}},\frac{y-b_{y}}{a_{y}}\right),$$

where *a* = (*a*_*x*_, *a*_*y*_) governs the scale and *b* = (*b*_*x*_, *b*_*y*_) the translation of the function. The values of *a* and *b* control the bandpass of the filter, generating highpass (H) or lowpass (L) filters.

The wavelet decomposition of an image consists of applying these wavelets horizontally and vertically, generating 4 images (LL, LH, HL, HH). The process is repeated iteratively on the LL subimage resulting in the standard pyramidal wavelet decomposition.

One of the key steps when using wavelets is the selection of the *mother wavelet*. There are numerous alternatives like Haar, Daubechies, or Symlet wavelets. In this paper, we used the Haar wavelets because they outperform the other wavelet families tested. Concretely, we applied a generalised Haar algorithm [17] using 2 scales, obtaining 8 result subimages.

The descriptor of an input image is constructed calculating the mean *μ* and the absolute average deviation *aad* of the input and LL images, and the energy *e* of the LH, HL, and HH images. Since we use 2 scales, our feature vectors contain 12 components.

#### 2.1.3. Co-Occurrence Features

Haralick et al. introduced co-occurrence features [19], a popular and effective texture descriptor based on the computation of the conditional joint probabilities of all pairwise combinations of grey levels, given an interpixel distance *d* and an orientation *θ*. This method generates a set of grey level co-occurrence matrices and extracts several statistics from their elements *P*_*θ*,*d*_(*i*, *j*).

**Figure 3:**
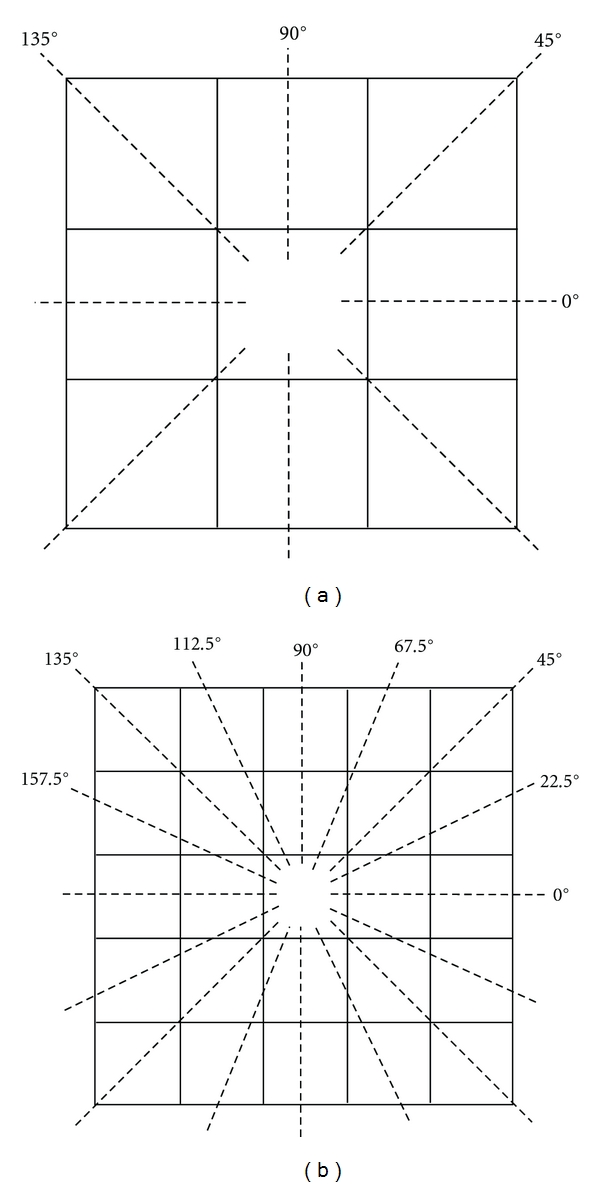
(a) Distance *d* = 1, 4 orientations. (b) Distance *d* = 2, 8 orientations.

For a distance *d* = 1, a total of 4 orientations must be considered (0°, 45°, 90° and 135°) and 4 matrices are generated (see Figure 3(a)). For a distance *d* > 1, the number of orientations increases and, therefore, so does the number of matrices. In general, the number of orientations for a distance *d* is 4*d*. As an example, Figure 3(b) depicts the orientations considered for the distance *d* = 2.

From each co-occurrence matrix, we compute a set of 14 statistics proposed by Haralick et al. in [19], representing features such as homogeneity or contrast. Next, we compute their mean and range across matrices obtaining a set of 28 features which will be the descriptor of the input image. 

#### 2.1.4. Markov Random Fields

Markov random fields (MRFs) are model based texture analysis methods that construct an image model whose parameters capture the essential perceived qualities of texture. An MRF [20] is a 2D lattice of points where each point is assigned a value that depends on its neighbouring values. Thus, MRFs generate a texture model by expressing the grey values of each pixel in an image as a function of the grey values in a neighbourhood of the pixel. 

Let *X*(*c*) be a random variable which denotes the grey value of the pixel *c* on an *N* × *M* image *I*, where *c* = 1,2, 3,…, *N* × *M*. Therefore, if *m* is a neighbour of *c*, *p*(*X*(*c*)) depends on *X*(*m*).

We need first to define the concept of neighbourhood as a previous step to create the MRF model. In this case, we consider the neighbourhood of a pixel as the set of pixels within a Chebyshev distance *d*. We have modelled the Markov process for textures using a Gaussian Markov random field defined as

(3) $$X\left(c\right)=\beta^{T}Q_{c}+e_{c},$$

where *e*_*c*_ is the zero mean Gaussian distributed noise and *β* coefficients describe the Markovian properties of the texture and the spatial interactions. Consequently, the *β* coefficients can be estimated using a least squares fitting.

In the present work, we have used the directional variances proposed by Çesmeli and Wang [21] to generate the image descriptor, defined as

(4) $$f_{i}=\frac{1}{N\times M}\underset{c\in I}{\sum }{\left[X\left(c\right)-\beta_{i}Q_{ci}\right]}^{2}.$$

For a distance *d*, the descriptor is composed of 4*d* features.

#### 2.1.5. Gabor Filters

Gabor filters are complex exponential signals modulated by Gaussians widely used in texture analysis. A two-dimensional Gabor filter [22], using cartesian coordinates in the spatial domain and polar coordinates in the frequency domain, is defined as

(5) $$g_{x_{0},y_{0},f_{0},\theta_{0}}=\exp\left\{i\left[2\pi f_{0}\left(x\cos\theta_{0}+y\sin\theta_{0}\right)+\phi \right]\right\}\text{gauss}\left(x,y\right),$$

where

(6) $$\begin{array}{cc} \text{gauss}\left(x,y\right) & =a\cdot \exp\{-\pi [a^{2}{\left(x\cos\theta_{0}+y\sin\theta_{0}\right)}^{2}\\ & \;+b^{2}{\left(x\sin\theta_{0}-y\cos\theta_{0}\right)}^{2}]\}, \end{array}$$

*a* and *b* model the shape of the filter, while *x*_0_, *y*_0_, *f*_0_, and *θ*_0_ represent the location in the spatial and frequency domains, respectively.

In the present work, we have created a bank of 16 Gabor filters centred at 4 frequencies and 4 orientations. The filter bank maps each input image to 16 result images, one per frequency-orientation pair.

Using the same idea as in *Butterworth Filters*, the descriptor of each output image is its uniform histogram with nonequidistant bins. Specifically, we have used 3, 5, 7, and 9 bin histograms as our feature vectors.

### 2.2. Colour Analysis

As previously mentioned, we have analysed both the texture and the colour of the tear film lipid layer. In the previous section, we introduced different texture extraction methods that operate in grayscale, after transforming the input image in RGB to grayscale. Now, we present two colour spaces and explain how the texture extraction methods operate in them.

The CIE 1976 L*a*b colour space [12] (Lab) is a chromatic colour space that describes all the colours that the human eye can perceive. Its use is recommended by CIE in images with natural illumination and its colorimetric components are differences of colours, which makes this colour space appropriate in texture extraction. In order to analyse the texture in this colour space, we transform the input image in RGB to the Lab colour space and analyse each component separately, generating three descriptors per image corresponding to the luminance component L, and the chromatic components *a* and *b*. Next, we concatenate these three descriptors to generate the final descriptor.

The RGB colour space [23] is an additive colour space defined by three chromaticities: red, green, and blue. It is not perceptually uniform and, in texture measuring, it is better to use differences of colours than the independent colours of the RGB model. Thus, to analyse this colour space, we have used the opponent process theory of human colour proposed by Hering [13]. This theory states that the human visual system interprets information about colour processing three opponent channels: red versus green, green versus red, and blue versus yellow. More precisely,

(7) $$\begin{array}{c} R_{G}=R-p*G,\\ G_{R}=G-p*R,\\ B_{Y}=B-p*\left(R+G\right), \end{array}$$

where *p* is a lowpass filter.

In the opponent colour space, we use the input image in RGB and calculate the three opponent channels, analysing the texture in each one separately. The final descriptor is the concatenation of the *R*_*G*_, *G*_*R*_, and *B*_*Y*_ descriptors.

### 2.3. Classification

Finally, we must classify the region of interest into one of the four categories proposed by Guillon. The classification task will be performed using five popular machine learning algorithms [24]: *Naive Bayes* (NB), a statistical classifier based on the Bayesian theorem and the maximum posteriori hypothesis that can predict class membership probabilities; *Logistic Model Tree* (LMT), an algorithm for supervised learning tasks which combines the logistic regression models with tree induction; *Random Tree* (RT), a tree drawn at random from a set of possible trees, where at random means that each tree in the set of the trees has an equal chance of being sampled; *Random Forest* (RF), a combination of tree predictors where each tree depends on the values of a random vector sampled independently and with the same distribution for all trees in the forest; *Support Vector Machine* (SVM) that based on the statistical learning theory performs classification by constructing an N-dimensional hyperplane that optimally separates the data in categories.

In Section 3, we will show the results, obtained by these algorithms, in terms of percentage accuracy. We will also compare the algorithms statistically, in order to determine which one performs best for the problem at hand.

## 3. Experimental Results

We have tested our methodology on a dataset composed of 105 images acquired from healthy patients with ages ranging from 19 to 33 years. These images have been annotated by optometrists from the School of Optics and Optometry of the Universidade de Santiago de Compostela. The dataset includes 29 open meshwork, 29 closed meshwork, 25 wave, and 22 colour fringe images. In order to analyse the generalisation of our results to larger dataset, a 10-fold cross-validation [25] has been performed.

In order to test the significance of the differences among classifier accuracies, we have performed several experiments with each texture analysis method using the five classifiers previously mentioned. The process is common in all the experiments: first, we applied the Lilliefors test for normality [26] and then an ANOVA test [27]. The ANOVA test compares the means of several distributions by estimating the variances among distributions and within a distribution. The null hypothesis, that all population means are equal, is tested and a *P*-value is computed. If the null hypothesis is rejected, we apply the Tukey's method, a multiple comparison procedure that tests all means pairwise to determine which ones are significantly different.

We have performed several experiments, the results of which appear in tables in terms of percentage accuracy. From top to bottom, each cell shows the results obtained in grayscale, Lab, and opponent colours. We have highlighted the best results in each colour space.

Our first experiment was performed using Butterworth filters and analysing each frequency band separately.  Table 1 shows the results obtained. The Lilliefors test for normality accepted the null hypothesis that the data came from a normal distribution in all the colour spaces. Therefore, we performed the ANOVA test obtaining the results depicted in Table 2. In grayscale, the ANOVA test rejected the null hypothesis and the Tukey's test concluded that there are significant differences among SVM, NB, and RT but not among SVM, LMT, and RF; so, SVM, LMT, and RF are the best classifiers in this case. Regarding Lab, the ANOVA test accepted the null hypothesis so no classifier performs significantly different from the others. Finally, the ANOVA test in opponent colours rejected the null hypothesis and the multiple comparison test concluded that there are significant differences among SVM and all the classifiers but LMT.

We did no experiment related to the discrete wavelet transform because there are not enough data to perform the statistical tests.  Table 3 shows the results obtained with all the classifiers using this method.

Our second experiment analyses the co-occurrence features and considers 7 distances separately, obtaining the results in Table 4. In the three colour spaces, the Lilliefors test accepted the null hypothesis and the ANOVA test rejected the null hypothesis in the three colour spaces, as Table 5 shows. The Tukey's test also concluded the same in the three colour spaces: there are significant differences among the SVM, which is the method that performs best, and the other four classifiers.

The next experiment consisted of analysing the Markov random fields method with 10 different neighbourhoods. Its results are depicted in Table 6. In grayscale, the Lilliefors test accepted the null hypothesis and the ANOVA test rejected it as we can see in Table 7. Finally, the multiple comparison test concluded that the SVM has significant differences with all the classifiers. In Lab and opponent colours, the results obtained with the NB classifier are not normally distributed. The NB classifier produced the poorer results in terms of percentage accuracy so we have eliminated it from the experiment. Using the other four classifiers, the ANOVA test produced the results in Table 7, rejecting the null hypothesis in both colour spaces. Finally, the multiple comparison test concluded that SVM has significant differences with the other classifiers.

Our last experiment analyses the Gabor filters using 4 different histogram sizes.  Table 8 shows its results in the three colour spaces. In grayscale, the Lilliefors test accepted the null hypothesis and then, the ANOVA test concluded that there are significant differences among the classifiers (see Table 9). Again, the SVM is significantly different from the others classifiers according to the Tukey's test. Regarding Lab, the Lilliefors test rejected the null hypothesis for the NB classifier, which was not included in the ANOVA test. As we can see in Table 9, the ANOVA test rejected the null hypothesis and the multiple comparison test selected the SVM as the classifier with significant differences with respect to the others. In opponent colours, the SVM did not pass the normality test and was not considered in the ANOVA test. This test concluded that there are significant differences among classifiers, as we can see in Table 9, and the Tukey's test selected the RF and LMT as the statistically different ones.

As a summary, Table 10 shows the best classifiers for each texture extraction method in the three colour spaces, according to the experiments performed. Analysing these results, we can see that SVM outperforms the other classifiers in most cases. This outperforming is because the SVM fits better the boundaries between classes.

Regarding colour analysis, the use of colour information improves the results compared to grayscale because some lipid layers contain not only morphological features, but also colour features. On the other hand, all texture analysis methods perform quite well providing results over 80% accuracy, but co-occurrence features generate the best result. Although Markov random fields use information of the pixel's neighbourhood, as the co-occurrence features do, this method does not work so well because the statistics proposed by Haralick et al. provide much more information. In short, the combination of co-occurrence features and the Lab colour space produces the best classification result with maximum accuracy over 96%. We should also consider Gabor filters because, in combination with the Lab colour space too, it is the second best method with maximum accuracy over 95% and it is computationally faster than co-occurrence features.

Finally, we would like to emphasise the clinical significance of these experimental results. Using the Tearscope, lipid layer thickness can be assessed based on interference phenomena produced over the whole surface. In [28], it was compared the performance of two observers with that obtained by an observer experienced in lipid layer pattern grading, designed as reference examiner. For thinner patterns (meshwork), observer 1 showed an agreement of 96% with the reference observer, whereas observer 2 showed an agreement of 91%. Better agreement with the reference observer was obtained for thicker patterns, easier than meshwork patterns; being 100% and 96% for observer 1 and observer 2, respectively. When considering colour fringe pattern, the agreement was even better, reaching a value of 100% for both observers. The results indicate that, after training, subjective observers can obtain good similarity among them. Therefore, although the Tearscope has proved its validity, some amount of training is needed to interpret the lipid layer patterns. This difficulty in interpreting the patterns and the lack of a huge bank of images for reference purposes has meant that many eye care professionals have abandoned this test. Our results show that it is possible to correctly categorise lipid layer patterns and eliminate the subjectivity of the test, through a completely automatic process which provides maximum accuracy over 95%.

## 4. Conclusions and Future Work

In this paper, we have presented a study of different machine learning algorithms to classify the tear film lipid layer, using the feature vectors extracted by different texture analysis methods in three colour spaces.

In general, the SVM classifier produces the best results independently of the texture extraction method and the colour space, compared with four other machine learning algorithms. The objective of this work was to show if the differences among classifiers were significant and we could establish SVM as the most suitable method. We first applied the Lilliefors test to assess the normality of the results in terms of percentage accuracy. Based on the conclusions of this test, we applied the ANOVA test in order to check whether the differences among classifiers were significant or not. If they were significant, the Tukey's test was applied to decide which classifier or classifiers were significantly different from the others.

The SVM classifier obtains the best results and is significantly different to the other classifiers so we should consider it in future works as the most competitive method. We should also consider the LMT because it is the second most competitive method according to the results obtained and it has an advantage compared to SVM: it does not need parameter tuning.

In many cases, the tear film lipid layer is very heterogeneous and makes its classification into a single Guillon category impossible. This heterogeneity is a sign of meibomian gland abnormality and leads us to our future work: performing local analysis and classifications, allowing the detection of several categories in a single photograph.

## Figures and Tables

**Table 1:** Butterworth filters: categorisation accuracy (%) in grayscale, Lab, and opponent colours using 16 bin histograms.

	Frequency bands									
Classifiers	1	2	3	4	5	6	7	8	9	Avg.
NB	50.48	**59.05**	65.71	60.00	59.05	55.24	48.57	46.67	43.81	54.29
	65.71	71.43	79.05	77.14	74.29	70.48	66.67	46.67	44.76	66.24
	59.05	60.95	57.14	57.14	59.05	53.33	50.48	48.57	44.76	54.50

LMT	62.86	53.33	58.10	62.86	64.76	**66.67**	58.10	54.29	43.81	58.31
	60.95	72.38	77.14	75.24	81.90	73.33	72.38	60.00	57.14	70.05
	58.10	54.29	66.67	76.19	74.29	61.90	64.76	58.10	51.43	62.86

RT	47.62	41.90	54.29	55.24	60.95	65.71	53.33	52.38	32.38	51.53
	48.57	65.71	75.24	75.24	67.62	72.38	67.62	50.48	45.71	63.17
	48.57	53.33	61.90	67.62	53.33	60.00	62.86	58.10	55.24	57.88

RF	42.86	48.57	62.86	60.00	66.67	64.76	60.00	50.48	48.57	58.09
	**63.81**	76.19	79.05	80.00	75.24	78.10	74.29	61.90	56.19	71.64
	54.29	65.71	68.57	65.71	68.57	69.52	61.90	53.33	57.14	62.75

SVM	**61.90**	57.14	**73.33**	**72.38**	**72.38**	**66.67**	**68.57**	**61.90**	**53.33**	**62.59**
	**63.81**	**80.95**	**85.71**	**88.57**	**89.52**	**80.00**	**75.24**	**64.76**	**70.48**	**77.67**
	**60.00**	**70.48**	**82.86**	**77.14**	**84.76**	**74.29**	**73.33**	**66.67**	**61.90**	**72.38**

**Table 2:** ANOVA results, Butterworth filters in the three colour spaces. SS: sum of squared deviations about the mean. df: degrees of freedom. MS: variance.

Grayscale					
Source	SS	df	MS	F	*P*-value
Between	976.06	4	244.02	3.74	<0.05
Within	2611.87	40	65.30		

Total	3587.93	44			

Lab					
Source	SS	df	MS	F	*P*-value

Between	1097.44	4	274.36	2.56	>0.05
Within	4287.01	40	107.18		

Total	5384.45	44			

Opponent colours					
Source	SS	df	MS	F	*P*-value

Between	1640.57	4	410.14	8.16	<0.05
Within	2009.93	40	50.25		

Total	3650.50	44			

**Table 3:** The discrete wavelet transform: categorisation accuracy (%) in grayscale, Lab, and opponent colours.

Classifiers	*μ* and *aad* of image and LL;
	*e* of LH, HL, and HH
NB	67.62
	66.67
	62.86

LMT	67.62
	80.95
	76.19

RT	73.33
	71.43
	65.71

RF	75.24
	86.67
	70.48

SVM	**85.71**
	**88.57**
	**84.76**

**Table 4:** Co-occurrence Features: categorisation accuracy (%) in grayscale, Lab, and opponent colours.

	Distances							
Classifiers	1	2	3	4	5	6	7	Avg.
NB	68.57	69.52	72.38	75.24	75.24	72.38	70.48	71.97
	75.24	83.81	81.90	83.81	85.71	86.67	86.67	83.40
	67.62	73.33	72.38	73.33	74.29	74.29	75.24	72.93

LMT	75.24	78.10	76.19	77.14	77.14	80.00	82.86	78.09
	80.00	82.86	80.00	83.81	86.67	86.67	82.86	83.27
	70.48	80.95	78.10	81.90	76.19	79.05	80.95	78.23

RT	74.29	63.81	75.24	74.29	78.10	71.43	71.43	72.66
	68.57	76.19	76.19	80.95	71.43	71.43	76.19	74.42
	63.81	65.71	74.29	64.76	71.43	77.14	62.86	68.57

RF	71.43	74.29	83.81	84.76	82.86	80.00	76.19	79.05
	83.81	87.62	81.90	88.57	86.67	90.48	83.81	86.12
	77.14	81.90	73.33	82.86	78.10	82.86	82.86	79.86

SVM	**80.00**	**84.76**	**87.62**	**89.52**	**91.43**	**90.48**	**92.38**	**88.03**
	**89.52**	**90.48**	**94.29**	**94.29**	**95.24**	**96.19**	**95.24**	**93.60**
	**85.71**	**89.52**	**90.48**	**89.52**	**90.48**	**90.48**	**91.43**	**89.66**

**Table 5:** ANOVA results, co-occurrence features in the three colour spaces. SS: sum of squared deviations about the mean. df: degrees of freedom. MS: variance.

Grayscale					
Source	SS	df	MS	F	*P*-value
Between	1165.72	4	291.43	18.29	<0.05
Within	477.98	30	15.93		

Total	1643.71	34			

Lab					
Source	SS	df	MS	F	*P*-value

Between	135.20	4	331.30	29.18	<0.05
Within	340.63	30	11.35		

Total	1665.83	34			

Opponent colours					
Source	SS	df	MS	F	*P*-value

Between	1778.14	4	444.53	31.13	<0.05
Within	428.42	30	14.28		

Total	2206.56	34			

**Table 6:** Markov random fields: categorisation accuracy (%) in grayscale, Lab, and opponent colours.

	Distances										
Classifiers	1	2	3	4	5	6	7	8	9	10	Avg.
NB	38.10	37.14	38.10	36.19	36.19	36.19	35.24	35.24	34.29	33.33	36.00
	45.71	42.86	39.05	31.43	31.43	30.48	32.38	31.43	30.48	30.48	34.57
	58.10	38.10	33.33	34.29	37.14	33.33	34.29	33.33	37.14	38.10	37.72

LMT	51.43	65.71	60.00	55.24	59.05	53.33	60.95	53.33	52.38	64.76	57.62
	59.05	60.00	67.62	62.86	67.62	60.00	60.00	51.43	49.52	55.24	59.33
	78.10	66.67	68.57	65.71	67.62	64.76	60.95	60.95	64.76	60.00	65.80

RT	52.38	55.24	52.38	47.62	54.29	55.24	57.14	52.38	54.29	60.00	54.10
	42.86	56.19	52.38	57.14	51.43	48.57	52.38	42.86	39.05	57.14	50.00
	67.62	58.10	57.14	52.38	59.05	52.38	49.52	50.48	50.48	58.10	55.23

RF	57.14	71.43	63.81	59.05	58.10	60.95	60.00	60.95	60.00	68.57	62.00
	51.43	59.05	66.67	55.24	60.00	55.24	60.95	61.90	55.24	62.86	58.89
	80.00	66.67	60.95	59.05	66.67	65.71	61.90	54.29	60.95	54.29	63.05

SVM	**61.90**	**78.10**	**78.10**	**83.81**	**81.90**	**80.00**	**75.24**	**76.19**	**73.33**	**77.14**	**76.57**
	**66.67**	**78.10**	**83.81**	**80.00**	**80.95**	**80.00**	**82.86**	**80.00**	**79.05**	**74.29**	**78.57**
	**84.76**	**80.00**	**84.76**	**82.86**	**80.00**	**84.76**	**77.14**	**80.00**	**76.19**	**73.33**	**80.38**

**Table 7:** ANOVA results, Markov random fields in the three colour spaces. SS: sum of squared deviations about the mean. df: degrees of freedom. MS: variance.

Grayscale					
Source	SS	df	MS	F	*P*-value
Between	8574.66	4	2143.67	109.52	<0.05
Within	880.82	45	19.57		

Total	9455.48	49			

Lab					
Source	SS	df	MS	F	*P*-value

Between	4352.63	3	1450.88	47.11	<0.05
Within	1108.76	36	30.8		

Total	5461.39	39			

Opponent colours					
Source	SS	df	MS	F	*P*-value

Between	3251.16	3	1083.72	33.36	<0.05
Within	1169.32	36	32.48		

Total	4420.47	39			

**Table 8:** Gabor Filters: categorisation accuracy (%) in grayscale, Lab and opponent colours.

	Number of bins				
Classifiers	3	5	7	9	Avg.
NB	60.00	59.05	58.10	60.00	59.29
	81.90	82.86	82.86	82.86	82.62
	62.86	60.00	62.86	64.76	62.62

LMT	80.95	77.14	74.29	75.24	76.91
	78.10	81.90	79.05	78.10	79.29
	70.48	71.43	71.43	79.05	73.10

RT	67.62	71.43	67.62	68.57	68.81
	73.33	80.95	68.57	65.71	72.14
	64.76	65.71	66.67	61.90	64.76

RF	73.33	66.67	72.38	69.52	70.48
	78.10	76.19	81.90	75.24	77.86
	78.10	72.38	70.48	80.00	75.24

SVM	**88.57**	**87.62**	**86.67**	**86.67**	**87.38**
	**92.38**	**94.29**	**95.24**	**95.24**	**94.29**
	**86.67**	**88.57**	**88.57**	**88.57**	**88.10**

**Table 9:** ANOVA results, Gabor filters in the three colour spaces. SS: sum of squared deviations about the mean. df: degrees of freedom. MS: variance.

Grayscale					
Source	SS	df	MS	F	*P*-value
Between	1732.62	4	433.15	95.67	<0.05
Within	67.92	15	4.53		

Total	1800.53	19			

Lab					
Between	1071.28	3	357.09	24.58	<0.05
Within	174.31	12	14.53		

Total	1245.59	15			

Opponent colours					
Between	457.58	3	152.52	13.68	<0.05
Within	133.81	12	11.15		

Total	591.39	15			

**Table 10:** Best classifiers using different texture extraction methods in the three colour spaces considered.

	Grayscale	Lab	Opponent colours
Butterworth filters	SVM, LMT, RF	No significant differences	SVM, LMT
The discrete wavelet transform	No data	No data	No data
Co-occurrence features	SVM	SVM	SVM
Markov random fields	SVM	SVM	SVM
Gabor filters	SVM	SVM	LMT, RF
